# A gated group sequential design for seamless Phase II/III trial with subpopulation selection

**DOI:** 10.1186/s12874-022-01825-0

**Published:** 2023-01-03

**Authors:** Guanhong Miao, Jason J. Z. Liao, Jing Yang, Keaven Anderson

**Affiliations:** 1 Department of Biostatistics, University of Florida, Gainesville, FL 32611 USA; 2 Biostatistics, Incyte Corporation, DE 19803 Willington, USA; 3 BARDS, Merck & Co., Inc, North Wales, PA 19454 USA

**Keywords:** Gated group sequential design, Seamless Phase II/III, Subpopulation selection, Power, Type I error

## Abstract

**Background:**

Due to the high cost and high failure rate of Phase III trials where a classical group sequential design (GSD) is usually used, seamless Phase II/III designs are more and more popular to improve trial efficiency. A potential attraction of Phase II/III design is to allow a randomized proof-of-concept stage prior to committing to the full cost of a Phase III trial. Population selection during the trial allows a trial to adapt and focus investment where it is most likely to provide patient benefit. Previous methods have been developed for this problem when there is a single primary endpoint and two possible populations.

**Methods:**

To find the population that potentially benefits with one or two primary endpoints (e.g., progression free survival (PFS), overall survival (OS)), we propose a gated group sequential design for a seamless Phase II/III trial design with adaptive population selection.

**Results:**

The investigated design controls the familywise error rate and allows multiple interim analyses to enable early stopping for efficacy or futility. Simulations and an illustrative example suggest that the proposed gated group sequential design has more power and requires less time and resources compared to the group sequential design and adaptive design.

**Conclusions:**

Combining the group sequential design and adaptive design, the gated group sequential design has more power and higher efficiency while controlling for the familywise error rate. It has the potential to save drug development cost and more quickly fulfill unmet medical needs.

## Background

The high failure rate of phase III trials combined with their substantial cost makes selecting an appropriate treatment and population for evaluation of paramount importance in drug development [1]. Seamless Phase II/III multi-arm clinical trials use the initial part of the trial (Phase II) to investigate all treatments and/or populations and an in-depth evaluation on the promising one(s) in the second part (Phase III). Using data accumulated across both phases of a single Phase II/III trial for inference enable more efficient and effective development of a treatment for an appropriate indication than separate trials for Phases II and III.

Considering a second line small cell lung cancer clinical trial, a platinum-sensitive sub-group yields a much greater treatment benefit. Even if the treatment benefit in the platinum-resistant sub-group is less certain, from a marketing perspective, the all-comer population with the inclusion of the platinum-resistant sub-group can give maximum patient benefit, followed by market value if the platinum-resistant sub-group also receives benefit from the experimental treatment. Under this circumstance, a direct Phase III trial with a broad population can be risky. A more efficient approach could be a seamless Phase II/III design with population selection in the Phase II portion of the trial followed by a potentially targeted Phase III enrollment with focused patient population to confirm the benefit. Benefit for either progression free survival (PFS) or overall survival (OS) could justify a new treatment paradigm. This is an extension of method for a single primary endpoint by Jenkins et al. [2].

In clinical trials, the clinical benefit of an intervention is often characterized by multiple outcomes. For multiple hypothesis testing problems, the familywise error rate (FWER), the probability of erroneously rejecting at least one null hypothesis, needs to be bounded by a pre-specified significance level α. A sequence of methods derived from weighted Bonferroni-based closed test procedures have been proposed to control the FWER for multiple testing. Examples of such methods include Bonferroni-Holm procedure [3], gatekeeping procedures based on Bonferroni adjustments [4] and the graphical approach [5, 6]. As group sequential designs are widely used and commonly employed in order to facilitate early efficacy testing, the application of group sequential designs to multiple endpoints becomes popular and has been widely studied recently [6–16].

Adaptive seamless Phase II/III designs allow Phase II assessment of whether within-trial extension to Phase III is justified. Here we consider that the adaptation includes choosing a meaningful population for an effective investment with high probability of success. A pre-defined, targeted sub-group and the full population are both studied in the first stage of the adaptive Phase II/III design. Investment in the second stage of the adaptive Phase II/III design is then focused on the population(s) most likely to provide patient benefit after the futility analysis at the end of Phase II. Due to the multiple sources potentially contributing to the decision error in this type of design, the FWER control should be studied carefully. The closed testing procedure [17] is usually applied to test multiple hypotheses in the setting of population selection. The FWER control strategies using multiple testing method [18, 19], combination test method [20, 21], the marginal *p*-value combinational approach [22], and a conditional error function approach [23] have been proposed. The application of adaptive Phase II/III designs to multiple endpoints has been investigated using different methods [2, 24–26].

To improve the trial efficiency in the adaptive phase II/III design, we propose a method to combine group sequential design (GSD) with the adaptive design. With the implement of GSD, the trial can stop early to save time and resources. However, the closed testing principle between the sub-group and the full population could dramatically decrease the power of an adaptive Phase II/III design when only one group has meaningful efficacy. To improve the power while controlling FWER, we propose a gated group sequential design (*g*GSD) combining the group sequential design and the adaptive design. The endpoints in the sub-group and full population are tested with a pre-specified order using the hierarchical testing [9]. Methods section illustrates the details of the proposed design. The performance of *g*GSD is evaluated by simulations, and an illustrative example is used to illustrate the design and its efficiency in Results section. The Summary section summarizes the proposed study design.

## Methods

We consider a randomized, parallel group clinical trial with two treatment arms – experimental and control, and dual primary endpoints – arbitrarily OS and PFS as a prototypical example. There is an interest to investigate the efficacy of the experimental treatment in both the full population (F) and a targeted sub-group (S). Four null hypotheses below are of interest:

1. ${H}_{0}^{\left\{F, OS\right\}}$: no difference in OS between arms in the full population;
2. ${H}_{0}^{\left\{F, PFS\right\}}$: no difference in PFS between arms in the full population;
3. ${H}_{0}^{\left\{S, OS\right\}}$: no difference in OS between arms in the targeted sub-group;
4. ${H}_{0}^{\left\{S, PFS\right\}}$: no difference in PFS between arms in the targeted sub-group.

**Fig. 1:**
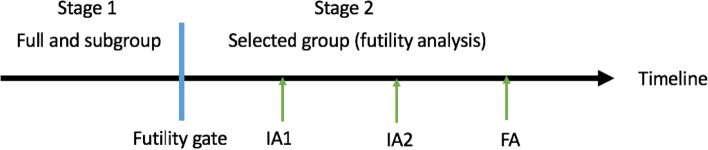
Analysis flowchart for the seamless Phase II/III design. IA: interim analysis; FA: final analysis

Let ${\alpha }_{1}$, ${\alpha }_{2}$, ${\alpha }_{3}$ and ${\alpha }_{4}$ be the initial significance level for the hypotheses ${H}_{0}^{\left\{F, OS\right\}}$, ${H}_{0}^{\left\{F, PFS\right\}}$, ${H}_{0}^{\left\{S, OS\right\}}$ and ${H}_{0}^{\left\{S, PFS\right\}}$, respectively, and $\mathrm{\alpha }$ be the overall significance level. Jenkins, et al. [2] proposed a method for population selection in the seamless adaptive design framework with only one analysis in stage 2 after population selection in stage 1. In this paper, we extend their method for population selection to control FWER for all four of the aforementioned hypotheses. We further add a group sequential design strategy in stage 2 for flexible early efficacy testing. The design consists of an initial learning stage (stage 1) analogous to a randomized Phase II trial and a second confirmatory phase (stage 2) analogous to a randomized Phase III trial. The selection between populations F and S is based on the PFS results at the end of stage 1. Based on that, the trial can either stop for futility, or continue to stage 2 in both populations F and S, or the sub-group S only, or the full population F only without analyzing the sub-group S in stage 2. Note that there is no hypothesis testing at the end of stage 1. In stage 2, we consider group sequential setting with $K-1$ interim analyses and one final analysis, where PFS and OS in populations F and/or S are tested by using group sequential approaches, with alpha allocation following the graphical approach [6]. Figure 1 shows the analysis flowchart for K = 3.

According to the FDA guidance for adaptive design [27], the design, conduct, and analysis of an adaptive clinical trial intended to provide substantial evidence of effectiveness should satisfy four key principles: 1) the chance of erroneous conclusions should be adequately controlled, 2) estimation of treatment effects should be sufficiently reliable, 3) details of the design should be completely pre-specified, and 4) trial integrity should be appropriately maintained. There are three potential reasons for inflation of Type I error: 1) early rejection of null hypothesis at interim analysis; 2) adaptation of design features and combination of information across trial stages; and 3) multiple hypothesis testing. To control the type I error rate, the following strategies are proposed: group sequential plans for early rejection; the combination of *p*-values using methods such as the inverse normal method for adaptation; multiple testing methodologies such as the closed testing procedures for multiple hypothesis. If needed, all three approaches can be combined to control the FWER.

For subjects recruited in stage 1, the nominal one-sided observed *p*-values of $H_0^{\left\{F,\cdot\right\}}$ and ${H}_{0}^{\left\{S, \cdot \right\}}$ at the *k*th analysis ($k=\mathrm{1,2},\dots ,K$) will be denoted by ${p}_{1k}^{\left\{F, \cdot \right\}}$ and ${p}_{1k}^{\left\{S, \cdot \right\}}$, respectively. For subjects recruited in stage 2, the nominal one-sided observed *p*-values of ${H}_{0}^{\left\{F, \cdot \right\}}$ and ${H}_{0}^{\left\{S, \cdot \right\}}$ at the *k*th analysis ($k=\mathrm{1,2},\dots ,K$) will be denoted by ${p}_{2k}^{\left\{F, \cdot \right\}}$ and ${p}_{2k}^{\left\{S, \cdot \right\}}.$ The goal is to control the FWER (i.e., the probability of rejecting at least one of the true null hypotheses ${H}_{0}^{\left\{F, OS\right\}}$, ${H}_{0}^{\left\{S, OS\right\}}$, ${H}_{0}^{\left\{F, PFS\right\}}$ and ${H}_{0}^{\left\{S, PFS\right\}}$) at a nominal level α. We consider all potential reasons of type I error inflation, with the closed testing principle applied for multiple testing, inverse combination testing used to analyze the data from two stages, and the graphical approach applied for group sequential analyses with different hypotheses. Combining these strategies, the FWER of the proposed design is strictly controlled [2, 6].

At the end of stage 1, the non-binding futility analysis for PFS in the sub-group *S* and full population *F* are performed. This determines whether the trial can continue to stage 2 with one or two populations, or just stop at the end of stage 1. No testing for rejection is done at the end of stage 1. Only one futility analysis is conducted no matter how many interim analyses might follow in the second stage, although additional futility analyses could be added as they only decrease Type I error. Let ${HR}^{F}$ and ${HR}^{S}$ be the estimated hazard ratio (HR) of the full population and the sub-group, and ${\theta }^{F}$ and ${\theta }^{S}$ be the pre-specified hazard ratio threshold for the full population and the sub-group, respectively. Table 1 provides the decision rule for population selection. We choose ${\theta }^{x}$ ($x=F,S$) to ensure that $\mathrm{P}\left(\mathrm{HR}>{\theta }^{x}|true HR\right)={\gamma }^{x}$ where ${\gamma }^{x}$ is a pre-specified threshold that the trial does not pass the futility gate under the true alternative HR. Under equal randomization, log(HR) approximately follows a normal distribution with mean log(true HR) and variance 4/(number of events). This gives a way to calculate the aforementioned thresholds.

**Table 1:** Population selection rule at the end of stage 1

	${HR}^{F}<{\theta }^{F}$	${HR}^{F}\ge {\theta }^{F}$
${HR}^{S}<{\theta }^{S}$	continue for F and S	continue for S only
${HR}^{S}\ge {\theta }^{S}$	continue for F only	stop for futility

### Stage 2

Once the futility boundary at the end of stage 1 is passed, the trial will continue to stage 2 with one or two populations. As described above, there are three possible scenarios in stage 2.

- *Scenario 1: continue to stage 2 in the sub-group S only with the planned sample size in S, allocating additional alpha to S*; i.e., ${\alpha }_{1}={\alpha }_{2}=0$*;*
- *Scenario 2: continue to stage 2 in the full population F with the planned sample size in F without further analysis of S, allowing additional allocation of alpha to F*; i.e., ${\alpha }_{3}={\alpha }_{4}=0$*;*
- *Scenario 3: continue to stage 2 in both populations F and S with the planned sample size, continuing testing in both populations.*

The gated group sequential design (*g*GSD*)* incorporates the hierarchical testing strategy and the group sequential design. The hierarchical testing strategy was proposed by Glimm et al. [9] for the ordered testing of endpoints such as PFS and OS with FWER controlled. In our study design, we modify their strategy to accommodate multiple testing scenarios with FWER controlled between populations; i.e., the hierarchical testing strategy is used for the ordered testing of populations.

In *scenario 1*, only PFS and OS in the sub-group *S* will be tested according to the alpha allocated using the graphical approach. An arbitrary alternative graphical approach could also be used: e.g., ${H}_{0}^{\left\{S, PFS\right\}}$ is first tested with the full significance level $\mathrm{\alpha }$, and the full $\mathrm{\alpha }$ will be passed to test ${H}_{0}^{\left\{S, OS\right\}}$ if and when ${H}_{0}^{\left\{S, PFS\right\}}$ is rejected using the overall hierarchical method of Glimm, et al. [9]. Note that the patients for the F minus S population enrolled in stage 1 will be followed continuously since the information from those patients is used in the closed testing procedure.

In *scenario 2,* only PFS and OS in the full population *F* will be tested according to the alpha allocated using the graphical approach; analogous to *Scenario 1,* an alternate graphical approach could also be used: e.g.$, {H}_{0}^{\left\{F, OS\right\}}$ will be tested at level $\mathrm{\alpha }$ only if ${H}_{0}^{\left\{F, PFS\right\}}$ is rejected (a hierarchical approach).

**Fig. 2:**
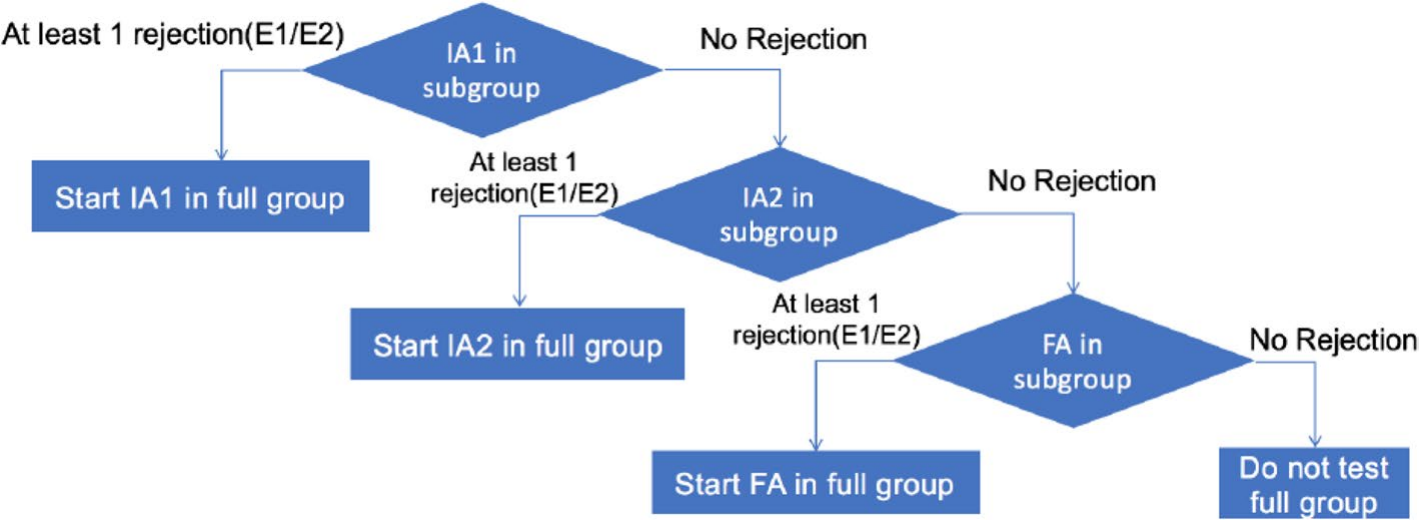
The testing procedures based on the gated group sequential design with K = 3 for Scenario 3 with both populations studied in stage 2. E: endpoint; IA: interim analysis; FA: final analysis

In *scenario 3,* the sub-group *S* and the full population *F* are tested hierarchically, i.e., the hypotheses in *F* will not be tested until at least one hypothesis in *S* is rejected. For the hypotheses within the same population *F* or *S*, the graphical approach of Maurer and Bretz [6] is applied. More specifically, the hypotheses in the sub-group *S* is tested based on the graphical approach with ${\alpha }_{3}+{\alpha }_{4}=\mathrm{\alpha }$. Under the hierarchical rule, the hypotheses in the full population *F* will be tested by using graphical approach with ${\alpha }_{1}+{\alpha }_{2}=\mathrm{\alpha }$ if at least one hypothesis in the sub-group *S* is rejected. The graphical approach ensures that $\mathrm{\alpha }$ reallocation occurs only between PFS and OS within the same group, and does not occur between different groups (i.e., between *F* and *S*). Note that the sequential testing rules and the timing of analyses is independent between the sub-group and the full population. Figure 2 illustrates the *g*GSD testing procedures in stage 2 for the efficacy analyses with *K* = 3. The design is event-driven and will continue to the final analysis unless all the hypotheses are rejected.

The inverse-normal combination test is applied to control the FWER regardless of the decision at the futility analysis at the end of stage 1. For the *k*-*th* analysis in stage 2, weights ${w}_{1k}$ and ${w}_{2k}$ are pre-specified to combine the *p*-values from stage 1 (${p}_{1k}$) and stage 2 (${p}_{2k}$), where ${w}_{1k}^{2}+{w}_{2k}^{2}=1$. The null hypothesis is rejected if ${w}_{1k}{\Phi }^{-1}\left(1-{p}_{1k}\right)+{w}_{2k}{\Phi }^{-1}\left(1-{p}_{2k}\right)\ge {c}_{k}$, where ${c}_{k}$ is the z-statistic boundary using the allocated alpha. It has been pointed out that the test statistics may not have the desired null distribution for time-to-event endpoints in a two-stage adaptive design [28, 29]. The violation of the independent increments assumption can lead to type I error inflation. To ensure that the hypothesis is tested with proper protection of the family-wise Type I error, we follow the method in previous adaptive design study [2]. Specifically, the *p*-values are calculated separately for subjects recruited to stage 1 (i.e., ${p}_{1k}$) and those recruited to stage 2 (i.e., ${p}_{2k}$). The additional follow-up of stage 1 subjects during stage 2 contributes to the stage 1 *p*-value (${p}_{1k}$). The closed testing procedures are applied to control the FWER. The Hochberg correction [30] with equal weighting, ${p}_{i}^{FS}=\mathrm{min}\left[2\mathrm{min}\left\{{p}_{i}^{F},{p}_{i}^{S}\right\},max\left\{{p}_{i}^{F},{p}_{i}^{S}\right\}\right]$, is used to compute the *p*-values of the intersection hypotheses between populations. The minimum z-statistic boundary of hypotheses with the allocated alpha in the intersection testing is used as ${c}_{k}$.

The weights and *p*-values to be used in combination tests are provided below, where the PFS endpoint is used as an example; the OS endpoint can be performed in a similar manner. Note that the weights ${w}_{1k}$ and ${w}_{2k}$ need to be pre-specified for controlling the FWER, and can be different for PFS and OS endpoints.

1. S only scenario – when considering ${H}_{0}^{\left\{S, PFS\right\}}$ only.
  Testing $${H}_{0}^{\left\{FS, PFS\right\}}$$:$${w}_{1k}{\Phi }^{-1}\left(1-{p}_{1k}^{\left\{FS,PFS\right\}}\right)+{w}_{2k}{\Phi }^{-1}\left(1-{p}_{2k}^{\left\{S,PFS\right\}}\right)$$;
  Testing $${H}_{0}^{\left\{S, PFS\right\}}$$: $${w}_{1k}{\Phi }^{-1}\left(1-{p}_{1k}^{\left\{S,PFS\right\}}\right)+{w}_{2k}{\Phi }^{-1}\left(1-{p}_{2k}^{\left\{S,PFS\right\}}\right)$$.
2. F only scenario—when considering ${H}_{0}^{\left\{F, PFS\right\}}$ only.
  Testing $${H}_{0}^{\left\{FS, PFS\right\}}$$:$${w}_{1k}{\Phi }^{-1}\left(1-{p}_{1k}^{\left\{FS,PFS\right\}}\right)+{w}_{2k}{\Phi }^{-1}\left(1-{p}_{2k}^{\left\{F,PFS\right\}}\right)$$;
  Testing $${H}_{0}^{\left\{F, PFS\right\}}$$: $${w}_{1k}{\Phi }^{-1}\left(1-{p}_{1k}^{\left\{F,PFS\right\}}\right)+{w}_{2k}{\Phi }^{-1}\left(1-{p}_{2k}^{\left\{F,PFS\right\}}\right)$$.
3. F and S scenario – when considering both ${H}_{0}^{\left\{F, PFS\right\}}$ and ${H}_{0}^{\left\{S, PFS\right\}}$.
  Testing $${H}_{0}^{\left\{FS, PFS\right\}}$$:$${w}_{1k}{\Phi }^{-1}\left(1-{p}_{1k}^{\left\{FS,PFS\right\}}\right)+{w}_{2k}{\Phi }^{-1}\left(1-{p}_{2k}^{\left\{FS,PFS\right\}}\right)$$;
  Testing $${H}_{0}^{\left\{F, PFS\right\}}$$: $${w}_{1k}{\Phi }^{-1}\left(1-{p}_{1k}^{\left\{F,PFS\right\}}\right)+{w}_{2k}{\Phi }^{-1}\left(1-{p}_{2k}^{\left\{F,PFS\right\}}\right)$$;
  Testing $${H}_{0}^{\left\{S, PFS\right\}}$$: $${w}_{1k}{\Phi }^{-1}\left(1-{p}_{1k}^{\left\{S,PFS\right\}}\right)+{w}_{2k}{\Phi }^{-1}\left(1-{p}_{2k}^{\left\{S,PFS\right\}}\right)$$.

## Results

### Simulations

To illustrate the performance of the proposed design in terms of type I error and power, we conduct simulations and compare the performance with the other two well-established approaches:

- Group sequential design (GSD): group sequential design for the 4 hypotheses of interest using the graphical approach of Maurer and Bretz [6] without any population or hypothesis adaptation.
- Adaptive design (AD): subpopulation selection is performed in the futility analysis. The overall significance level is set to be $\mathrm{\alpha }$ to test all 4 hypotheses rather than setting the overall significance level to be α to test only 2 hypotheses in each population (S and F) in gGSD. The same alpha reallocation strategy [6] is used to control the FWER.

The gGSD is a seamless phase II/III trial integrating AD and GSD into one study design. Briefly, AD is implemented in the subgroup selection stage (futility analysis), followed by GSD in the second stage (i.e., two interim analyses and one final analysis). Three simulation settings are considered. Table 2 gives the detailed information for these three settings. In each setting, two interim analyses and one final analysis are planned in stage 2. Specifically, PFS testing is planned at IA1 and IA2 (which is also the final for PFS), while OS testing is planned at IA1, IA2 and FA. Some parameters are set to be the same for all three settings: 1) for the control arm, the median PFS (OS) is assumed to be 4 (10.5) months and 3 (5.7) months both in the sub-group and the complement of the sub-group, respectively; 2) the yearly dropout rates for PFS and OS are 10% and 1%, respectively. In settings 1 and 2, the hazard ratio (experimental/control) for PFS and OS are 0.7 for both the sub-group and the full population. In setting 3, the hazard ratios of PFS and OS are 0.7 for the sub-group, but 1 for the full population. For the full population: at the design stage, the information fractions for PFS are approximately 90% for IA1 and IA2 is the final analysis; the information fractions for OS are approximately 69% for IA1 and 92% for IA2. For the sub-group population: at the design stage, the information fraction for PFS is approximately 89% for IA1 and IA2 is the final analysis; the information fractions for OS are approximately 66% for IA1 and 91% for IA2. Some other parameters used in the simulations are provided in Table 2 below where the sample size is calculated based on the group sequential design with a power of at least 85% for all four hypotheses. The alpha boundaries are computed using the Lan-DeMets spending function approximating O'Brien-Fleming bounds with a total of 1-sided $\mathrm{\alpha }$=0.025.

**Table 2:** Parameters for three simulation settings

Setting	Sample Size	Subgroup Proportion	Enrollment Duration	Futility boundary	Dropout rate (yearly)	Median survival time	HR^b^	Design	Full Group		Sub-Group	
									${\alpha }_{1}$ (OS)	${\alpha }_{2}$ (PFS)	${\alpha }_{3}$ (OS)	${\alpha }_{4}$(PFS)
1	554	0.75	28 months	0.9 (SG^a^)	10% (PFS)	4 (PFS SG) 3 (PFS NSG^a^)	0.7	GSD & AD	0.22%	0.165%	1.28%	0.835%
				0.85 (FG^a^)	1% (OS)	10.5 (OS SG) 5.7 (OS NSG)		*g*GSD	1.429%	1.071%	1.513%	0.987%
2 & 3	924	0.5	33 months	0.85 (SG^a^)	10% (PFS)	4 (PFS SG) 3 (PFS NSG)	0.7^c^	GSD & AD	0.025%	0.017%	1.458%	1.00%
				0.83 (FG^a^)	1% (OS)	10.5 (OS SG) 5.7 (OS NSG)		*g*GSD	1.488%	1.012%	1.48%	1.02%

^a^*SG* Subgroup, *FG* Full group, *NSG* Complementary ofsub-group

^b^Hazard ratio (HR) is set to be the same for PFS, OS and sub-group, complementary of sub-group

^c^HR = 1 for full group in setting 3. Complementary of sub-group is defined as the full group excluding the sub-group

For each setting, the performance of GSD is provided as a reference for comparison. For AD and *g*GSD, the futility analyses for PFS are performed at the end of stage 1. This determines whether the trial continues to stage 2 with one or two populations, or the trial stops. Let the futility threshold ($\gamma$), the probability of the trial not passing the futility gate under the alternate hypothesis, be 5%. This results in ${\theta }^{F}=0.85$ and ${\theta }^{S}=0.9$ for setting 1, and ${\theta }^{F}=0.83$ and ${\theta }^{S}=0.85$ for settings 2 and 3.

The time-to-event data were generated using an R-package “simtrial” [31] with settings specified in Table 2. The “simtrial” package generates independent time-to-event datasets according to a user-specified trial design. Information of the enrollment, dropout, and infection processes are prespecified in each treatment arm. A total of 10,000 replications were performed for each setting. For AD and *g*GSD, eight different sets of weights were evaluated for the inverse-normal combination tests. Ideally, weights ${w}_{1k}$ and ${w}_{2k}$ would be chosen to be proportional to the square root of the number of events in each stage for the *k*-th analysis. As an example, set $\left(w_{1k}^{PFS},w_{2k}^{PFS}\right)$ = $\left(\sqrt{\frac{n_{1k,PFS}}{n_{1k,PFS}+n_{2k,PFS}}},\sqrt{\frac{n_{2k,PFS}}{n_{1k,PFS}+n_{2k,PFS}}}\right)$ for PFS hypothesis where ${n}_{ik, PFS}$ is the number of PFS events from stage i subjects (i = 1,2) and $\left(w_{1k}^{OS},w_{2k}^{OS}\right)$ = $\left(\sqrt{\frac{n_{1k,OS}}{n_{1k,OS}+n_{2k,OS}}},\sqrt{\frac{n_{2k,OS}}{n_{1k,OS}+n_{2k,OS}}}\right)$ for OS hypothesis where ${n}_{ik, OS}$ is the number of OS events from stage *i* subjects (*i* = 1,2). ${w}_{1k}$ and ${w}_{2k}$ need to be pre-specified in order to control the Type-I error rate. Since it is impossible to know the decision at futility analysis and the number of events from stage 1 and 2 for each efficacy analysis, we use pre-specified weights to compute *p*-values.

The proposed *g*GSD is FWER controlled and the simulations showed that it is conservative: e.g., the type I error is less than the specified 0.025 level as shown in Table 3. Table 4 shows the power of rejecting the sub-group (S), or both sub-group and full population (S&F). The performance of the proposed *g*GSD depends on the choice of the weights ${w}_{1k}$ and ${w}_{2k}$. The first set of weights are computed using the number of PFS/OS events in the simulation and are used as a reference. When ${w}_{1k}<{w}_{2k}$, AD and *g*GSD have lower power to detect treatment efficacy compared with GSD. When ${w}_{1k}\ge {w}_{2k}$, *gGSD* has higher power than GSD and AD. Table 4 indicates that the events driven weight or more weights for stage 1 data lead to a better *g*GSD performance. The performance of *g*GSD is robust for the weights as long as more weight is assigned to stage 1 data. Thus, assigning more weights for data from stage 1 is recommended in order to utilize the information more efficiently. The simulation results for setting 3 (only sub-group has significant treatment benefit) demonstrate that the proposed gGSD reduces the patient’s exposure to less effective treatment comparing to GSD if the complementary sub-group has less significant treatment effect since gGSD does not enroll patients in the complementary sub-group in stage 2.

**Table 3:** Family-wise error rate for three simulation settings

Weight(${w}_{1k},{w}_{2k}$)		Design	Setting 1	Setting 2/3
PFS	OS	GSD	0.014	0.017
(${w}_{1k}^{PFS},{w}_{2k}^{PFS}$)^a^	(${w}_{1k}^{OS},{w}_{2k}^{OS}$)^a^	AD	0.009	0.011
		*g*GSD	0.011	0.012
($\sqrt{0.2},\sqrt{0.8}$)	($\sqrt{0.2},\sqrt{0.8}$)	AD	0.007	0.009
		*g*GSD	0.010	0.011
($\sqrt{0.3},\sqrt{0.7}$)	($\sqrt{0.3},\sqrt{0.7}$)	AD	0.008	0.010
		*g*GSD	0.010	0.012
($\sqrt{0.5},\sqrt{0.5}$)	($\sqrt{0.5},\sqrt{0.5}$)	AD	0.009	0.011
		*g*GSD	0.011	0.013
($\sqrt{0.5},\sqrt{0.5}$)	($\sqrt{0.7},\sqrt{0.3}$)	AD	0.009	0.011
		*g*GSD	0.012	0.013
($\sqrt{0.7},\sqrt{0.3}$)	($\sqrt{0.7},\sqrt{0.3}$)	AD	0.009	0.011
		*g*GSD	0.011	0.012
($\sqrt{0.8},\sqrt{0.2}$)	($\sqrt{0.8},\sqrt{0.2}$)	AD	0.009	0.011
		*g*GSD	0.011	0.012
($\sqrt{0.6},\sqrt{0.4}$)	($\sqrt{0.6},\sqrt{0.4}$)	AD	0.009	0.011
		*g*GSD	0.011	0.013

^a^Weights defined in Simulations section (4^th^ paragraph) based on observed interim events and planned final events

**Table 4:** Power for three simulation settings

			Setting 1		Setting 2		Setting 3
Weight(${w}_{1k},{w}_{2k}$)		Design	S	S & F	S	S & F	S
PFS	OS	GSD	0.877	0.873	0.908	0.908	0.908
(${w}_{1k}^{PFS},{w}_{2k}^{PFS}$)^a^	(${w}_{1k}^{OS},{w}_{2k}^{OS}$)^a^	AD	0.903	0.914	0.945	0.947	0.908
		*g*GSD	0.914	0.925	0.945	0.947	0.908
($\sqrt{0.2},\sqrt{0.8}$)	($\sqrt{0.2},\sqrt{0.8}$)	AD	0.749	0.750	0.830	0.830	0.798
		*g*GSD	0.769	0.770	0.829	0.829	0.798
($\sqrt{0.3},\sqrt{0.7}$)	($\sqrt{0.3},\sqrt{0.7}$)	AD	0.808	0.813	0.877	0.878	0.844
		*g*GSD	0.826	0.830	0.877	0.878	0.846
($\sqrt{0.5},\sqrt{0.5}$)	($\sqrt{0.5},\sqrt{0.5}$)	AD	0.873	0.881	0.928	0.930	0.895
		*g*GSD	0.888	0.895	0.929	0.930	0.895
($\sqrt{0.5},\sqrt{0.5}$)	($\sqrt{0.7},\sqrt{0.3}$)	AD	0.894	0.903	0.939	0.941	0.904
		*g*GSD	0.908	0.916	0.938	0.940	0.904
($\sqrt{0.7},\sqrt{0.3}$)	($\sqrt{0.7},\sqrt{0.3}$)	AD	0.900	0.910	0.945	0.947	0.908
		*g*GSD	0.913	0.922	0.945	0.947	0.909
($\sqrt{0.8},\sqrt{0.2}$)	($\sqrt{0.8},\sqrt{0.2}$)	AD	0.903	0.914	0.945	0.948	0.905
		*g*GSD	0.915	0.925	0.946	0.948	0.906
($\sqrt{0.6},\sqrt{0.4}$)	($\sqrt{0.6},\sqrt{0.4}$)	AD	0.890	0.899	0.939	0.941	0.903
		*g*GSD	0.904	0.913	0.939	0.941	0.904

^a^Weights defined in early text based on observed interim events and planned final events

The power for sub-group (S) is calculated among 10,000 simulations with the sub-group passes the futility boundary. Similarly, the power for sub-group and full group (S &F) is calculated among 10,000 simulations with both the sub-group and full group pass the futility boundary

**Fig. 3:**
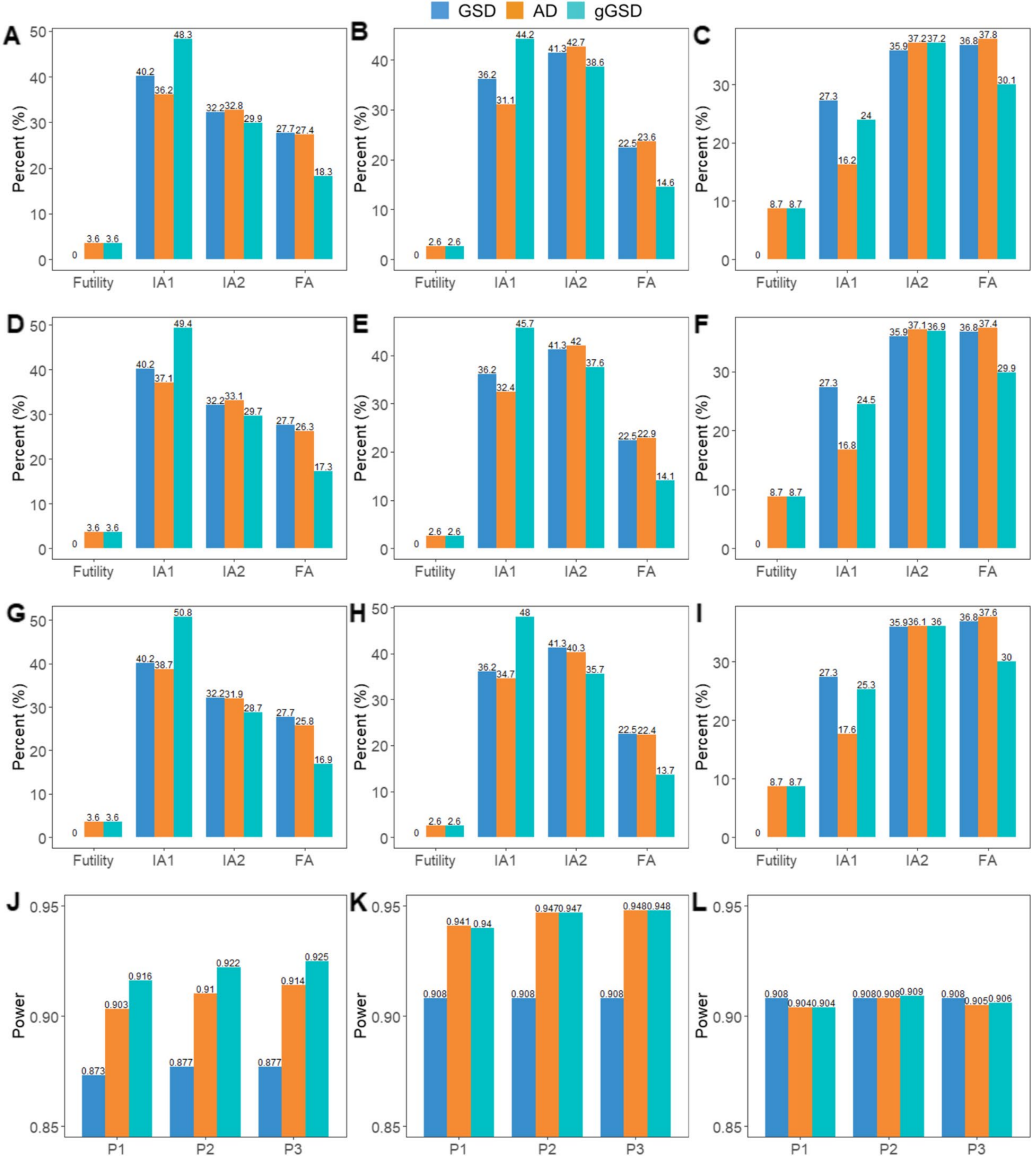
The stopping time (panels **A**-**I**) and the corresponding power (panels **J**-**L**) among 10,000 trials for three simulation settings. Only simulation results of three weight sets (P1: ($\sqrt{0.5},\sqrt{0.5}$) for PFS hypotheses and ($\sqrt{0.7},\sqrt{0.3}$) for OS hypotheses; P2: ($\sqrt{0.7},\sqrt{0.3}$) for both PFS and OS hypotheses; P3: ($\sqrt{0.8},\sqrt{0.2}$) for both PFS and OS hypotheses) with the highest power are shown in the figure. Panels **A**/**D**/**G**/**J**: setting 1; Panels **B**/**E**/**H**/**K**: setting 2; Panels **C**/**F**/**I**/**L**: setting 3; Panels **A**-**C**: stopping time for P1; Panels **D**-**F**: stopping time for P2; Panels **G**-**I**: stopping time for P3. The power for each setting under different weight sets is summarized in (panels **J**-**L**)

Another advantage of the proposed *g*GSD is that it can terminate early with high power. Figure 3 shows the stopping time of three designs for three weight sets with the highest power in Table 4. For GSD, the trial stops early if and only if all the four hypotheses are rejected before the final analysis. For example, there are 3 hypotheses being rejected in IA1 and the last hypothesis is rejected in IA2, then the termination point for this trial is at IA2. For AD and *g*GSD, the trial stops early if no sub-group/full group passes the futility boundary or all the hypotheses tested are rejected before the final analysis. Detailed requirements for early stopping of the trial are listed in Table 5. As shown in Fig. 3, gGSD is more efficient (i.e., higher probability to reject all the hypotheses tested and stop early before the final analysis) with higher or comparable power compared to GSD and AD (Fig. 3 panels J-L). Therefore *g*GSD requires less time and resources to prove new treatment efficacy than GSD and AD without sacrificing power for an important underlying benefit.

**Table 5:** Trial stopping point and requirements

Stopping point	GSD	AD/gGSD
Futility	–	Both sub-group and full group do not pass the futility boundary
IA1	All the 4 hypotheses are rejected at IA1	**(1)** At least one group passes the futility boundary. **(2)** All the hypotheses tested are rejected at IA1
IA2	**(1)** At least 1 hypothesis is not rejected at IA1. (**2)** All the 4 hypotheses are rejected at IA2	**(1)** At least one group passes the futility boundary. **(2)** At least 1 hypothesis is not rejected at IA1. **(3)** All the hypotheses tested are rejected at IA2
FA	**(1)** At least 1 hypothesis is not rejected at IA1 and IA2. (**2)** The trial stops at FA regardless of the number of hypotheses rejected	**(1)** At least one group passes the futility boundary. **(2)** At least 1 hypothesis is not rejected at IA1 and IA2. **(3)** The trial stops at FA regardless of the number of hypotheses rejected

### An illustrative example

We use an example with specified *p*-values to illustrate the potential advantage of the proposed *g*GSD compared to GSD. Consider a group sequential design for a Phase III 2^nd^ line small cell lung cancer trial with a 50% prevalence of platinum-sensitive subgroup where PFS and OS are the dual primary endpoints. This example contains a total of 924 patients with other parameters same as setting 2 listed in Table 2. The graphical approach [6] was used to control FWER of the four hypotheses with a total of FWER level 0.025. PFS and OS hypotheses are tested in two interim analyses and only OS hypotheses are tested in the final analysis.

This example illustrates that *g*GSD has more power to reject the null hypotheses compared to GSD. Table 6 contains the nominal *p*-values and data generated *p*-values at each interim analysis and the final analysis for GSD and *g*GSD. As shown in Table 6, none of the four hypotheses are rejected by using GSD. Using the *g*GSD and the gated rules in Table 1 with ${\theta }^{F}=0.83$ and ${\theta }^{S}=0.85$, stage 2 is continued for the full group only (i.e., Scenario 2 in stage 2). Once ${H}_{0}^{\left\{F, PFS\right\}}$ is rejected, ${H}_{0}^{\left\{F, OS\right\}}$ will be tested at level $\mathrm{\alpha }=0.025$. A fixed weight ${w}_{1k}$=${w}_{2k}$=$\sqrt{0.5}$ is used for all the *p*-value combination tests in *g*GSD. With a *p*-value of 0.0022 at the IA1, the PFS is rejected. A *p*-value of 0.0125 at IA1 fails to reject OS at IA1. Then the trial continues to IA2 for OS testing in the full group. With a *p*-value of 0.0019 at the IA2, the OS hypothesis is rejected at IA2. So none of the four hypotheses are rejected in GSD while *g*GSD rejects two full group hypotheses.

**Table 6:** Theoretical and specified parameters for the illustrative example

		GSD				*g*GSD			
		Subgroup		Full Group		Subgroup		Full Group	
Analysis	Boundary & *p*-value	PFS	OS	PFS	OS	PFS	OS	PFS	OS
IA1	Nominal *p*-value boundary	0.0036	0.0017	< 0.0001	< 0.0001	0.0037	0.0017	0.0039	0.0024
	Data generated *p*-value	0.0177	0.1205	0.0008	0.0104	–	–	**0.0022**	0.0125
IA2	Nominal *p*-value boundary	0.0088	0.0078	0.0002	0.0001	0.0090	0.0079	0.0089	0.0088
	Data generated *p*-value	0.0137	0.0502	0.00022	0.0023	–	–	–	**0.0019**
FA	Nominal *p*-value boundary		0.0120		0.0002		0.0122		0.0119
	Data generated *p*-value		0.0534		0.0011		–		–

The nominal *p*-value is the *p*-value boundary in a typical group sequential design under the allocated alpha in different IA time

The data generated *p*-value is the *p*-value from the test using the trial data

## Summary

Seamless Phase II/III designs are getting more attention and being increasingly adopted as a cost effective and time saving drug development strategy. In this paper, we proposed a gated group sequential design for seamless Phase II/III trial with potential sub-group selection. Combining this with GSD, our proposed *g*GSD design enables population selection and multiple interim analyses to enable early stopping. In this paper, we extended Jenkins, et al. [2] method for population selection to control FWER for all four of the aforementioned hypotheses with dual primary endpoints. The hierarchical testing strategy proposed by Glimm et al. [9] was modified to accommodate our multiple testing scenarios with FWER controlled between populations. Within each population, the graphical approach combined with standard group sequential design was used for flexibility. The familywise error rate of proposed *g*GSD is strictly controlled. A pre-specified sub-group and the full population are tested hierarchically to control the FWER. Simulation results and the illustrative example suggest that the gated group sequential design can reduce sample size compared to the other trial designs; e.g., the proposed gated group sequential design could achieve the same power with a smaller sample size compared to the commonly used GSD. Furthermore, the trial can terminate early with sufficient strong evidence from efficacy analyses and potentially moves efficacious products into market faster for unmet medical needs. A special note on the particular advantage of the gGSD over GSD in the simulation study occurs when the true benefit is in the sub-group, but not in the full group. The gGSD is designed to focus on the stage 2 selected population, increases power over a Phase III study of both populations and reduces the patient’s exposure to less effective treatment comparing to GSD if the complementary sub-group has less significant treatment effect.

The idea proposed in this paper can also be applied to conduct efficient trials and simultaneously investigate several vital questions for drug development, such as identifying the most beneficial sub-group for a new treatment or dose (treatment) selection problem. Moreover, the proposed *g*GSD is applicable to more than one sub-group where the sub-groups are nested. In this paper, the sub-group was pre-specified. However, this sub-group information may not be always accurately identified before the trial. Freidlin and Simon [32] proposed an adaptive signature design to find sensitive patients, without pre-specified, into a formal Phase III trial. The sub-group size does not have any impact on the method proposed in this paper. However, practically speaking, generally the sub-group size should be at least 50% of the full population to be financially feasible and maybe ethical reasonable for using this type of seamless design. The proposed seamless design shares the same potential operational challenges discussed in the literature that the trial team may choose to hold the enrollment while the team decides the population selection at the end of stage 1. Different approaches could be used in setting up the criteria for moving into stage 2. One such example could be the predictive probability as used in the Belle 4 study [33]. In an adaptive time-to-event design, the number of events collected in stage 2 could be influenced by a subpopulation selection. These issues arise from the fact that patients who are recruited before an interim analysis and hence enter the interim analysis as censored observations at the time of the interim can have an event later and then enter the analysis again. The strategy discussed in Jenkins et al. [2] could be used to address the independent increments assumption.

In this paper, the PFS of the dual-primary endpoints was used for the adaptation. Other surrogate “proof-of-concept” endpoint such as the objective response could be used if more appropriate. The *g*GSD is a two-stage trial design with two arms where the second stage data are used for a classical group sequential design framework. In this regard, the more commonly discussed multi-arm multi-stage (MAMS) design can be combined with *g*GSD. The research is under investigation. When there is a severe non-proportional hazard such as the delayed effect, the proposed gGSD in current format may be less efficient due to the potential poor performance in the futility analysis.

## Data Availability

All data used were simulated. The simulation programs can be accessed from the GitHub repository (https://github.com/populus-0112/gated-group-sequential-design_gGSD).
